# Tracking individual seed fate confirms mainly antagonistic interactions between rodents and European beech

**DOI:** 10.1098/rsbl.2024.0586

**Published:** 2025-01-22

**Authors:** Frederik Sachser, Georg Gratzer, Mario B. Pesendorfer, Heino Konrad, Iris Kempter, Ursula Nopp-Mayr

**Affiliations:** ^1^ Department of Ecosystem Management, Climate, and Biodiversity, Institute of Wildlife Biology and Game Management, BOKU – University of Natural Resources and Life Sciences, Vienna, Austria; ^2^ Department of Ecosystem Management, Climate, and Biodiversity, Institute of Forest Ecology, BOKU – University of Natural Resources and Life Sciences, Vienna, Austria; ^3^ Department of Forest Biodiversity and Nature Conservation, Austrian Research Centre for Forests, Vienna, Austria; ^4^ Migratory Bird Center, Smithsonian Conservation Biology Institute, DC, USA; ^5^ via donau – Österreichische Wasserstraßen-Gesellschaft mbH, Donau-City-Straße 1, Vienna 1220, Austria

**Keywords:** mast seeding, seed survival, seed dispersal, *Fagus sylvatica*, mutualism–antagonism continuum, synzoochory

## Abstract

Food-hoarding granivores act as both predators and dispersers of plant seeds, resulting in facultative species interactions along a mutualism–antagonism continuum. The position along this continuum is determined by the positive and negative interactions that vary with the ratio between seed availability and animal abundance, particularly for mast-seeding species with interannual variation and spatial synchrony of seed production. Empirical data on the entire fate of seeds up to germination and the influence of rodents on seed survival is rare, resulting in a lack of consensus on their position along the mutualism–antagonism continuum. Here, we quantified annual seed rain and rodent abundance in an old-growth European beech forest and tracked 639 beechnuts to the seedling stage with 84% of seeds successfully located. Over 4 study years that covered the range of seed-to-rodent ratios, not a single seed successfully germinated after dispersal, illustrating a predominantly antagonistic interaction between rodents and seeds of European beech. Therefore, our findings do not support the predator dispersal hypothesis and partially contradict the predator satiation hypothesis, as the highest number of germinants and intact seeds were found *in situ* after an intermediate seed crop, not a bumper crop. Our results underline the necessity to track seeds up to germination.

## 1. Introduction

Food-hoarding of seeds by animals is a globally pervasive species interaction whose outcome is context-dependent, falling along a continuum between antagonism and mutualism, as it can result in predation or seed dispersal (synzoochory) [1]. The relative position along the continuum is determined by the relative frequency of positive and negative interactions, which can vary due to physical and chemical characteristics of seeds, as well as the ratio between seed availability and animal abundance [2–5]. From the plant perspective, dispersal can improve fitness by reducing distance- or density-dependent effects of predators and pathogens, enable the colonization of suitable sites, or result in arrival at favourable sites that enhance survival of the seedling [6,7]. In synzoochory, these benefits are balanced by the costs of seed losses through predation or deposition at inadequate locations for germination, emergence and establishment [8]. Furthermore, for plant species with high interannual variation and spatial synchrony in seed production (masting), the outcome of the interactions varies year by year, complicating the quantitative assessment of the net effects of the species interaction on seed fate [9,10]. Even for plants with well-studied animal partners, seed fate is difficult to determine and often only estimated indirectly using statistical or mathematical models [11,12], as empirical data rarely cover the whole range of contexts in animal and seed abundances [5,13]. Furthermore, because many synzoochorous interactions cannot be observed directly, but rather require physical tracking or telemetry, determining the effects of handling by seed-hoarding animals is a veritable challenge [14]. Consequently, there is still a lack of consensus about the relative position of common food-hoarding species along the mutualism–antagonism spectrum [13,15].

Historically, rodents were predominantly considered plant antagonists, as they are effective post-dispersal predators [16], particularly in arid areas [17–19]. However, a growing body of literature reported potential seed dispersal benefits from rodent caching behaviour in temperate forests of Eurasia [20,21], North America [9,22,23] and South America [24]. The complexity of this plant–animal interaction prevents simple and generalizable conclusions and studies about synzoochory often use different methods to collect and analyse data, resulting in debates about model assumptions and validity or generality of conclusions [13,15,25]. While the vast majority of studies on seed handling by rodents investigate removal and caching, few provide direct evidence of dispersal resulting in germination or recruitment of seedlings [7,26].

We conducted a multi-year study to evaluate the net outcome of the interaction between granivorous rodents and European beech *Fagus sylvatica* in a primary old-growth forest. Supported by high-resolution long-term monitoring of seed rain [27] and rodent abundance dynamics [28], we used cafeteria experiments and tracking of seeds until germination [14] to determine the annual outcome of seed predation and dispersal interactions across the whole range of seed-to-predator ratios. The aim was to test prevailing hypotheses on the relationship between annual seed production and seed fate in masting species. Under the *predator satiation hypothesis*, the proportion of seeds that germinate after pre- and post-dispersal predation is predicted to increase with annual seed production levels as seed predators are overwhelmed by seed abundance [10,29,30]. The non-mutually exclusive *predator dispersal hypothesis* predicts that dispersal benefits, such as rate or distance, conferred by seed-hoarding animals increase in years of high seed production [9,31,32]. Alternatively, annual variation in seed crops may not result in benefits related to seed predation and dispersal by rodents, due to a lack of satiation, a hypothesis that was recently supported in a global meta-analysis [10]. Previous observations at our field site suggested such an effect, as we observed a complete lack of beech regeneration after a pronounced mast year in 2011 [33], and we are aware of a similar observation in another primary old growth forest in Poland after a mast event in 2003 (J Szwagrzyk 2004, unpublished data). We therefore hypothesized that rodents either consume seeds directly or disperse and cache them at inadequate places resulting in a reduced amount of germinants, placing this species interaction on the antagonistic part of the spectrum.

## 2. Material and methods

### (a) Study area and species

Rothwald is a strictly protected (IUCN cat. 1 a), never-logged primeval forest, located at the northern Limestone Alps of Austria (47°48′ to 47°45′ N, 15°01′ to 15°07′ E). Stands are dominated by European beech, with lower densities of co-dominant Norway spruce (*Picea abies*), and European silver fir (*Abies alba*), forming a two-layered canopy (see electronic supplementary material, S1 for a detailed description of the study area). The small mammal community mainly consists of several wood mouse species, which are difficult to discern under field conditions (*Apodemus flavicollis*, *A. sylvaticus* and *A. alpicola*, hereafter ‘*Apodemus* spp.’), as well as bank voles (*Myodes glareolus*) and edible dormice (*Glis glis*) [28,33].

### (b) Monitoring of seed rain and small mammal abundance

**Figure 1.**
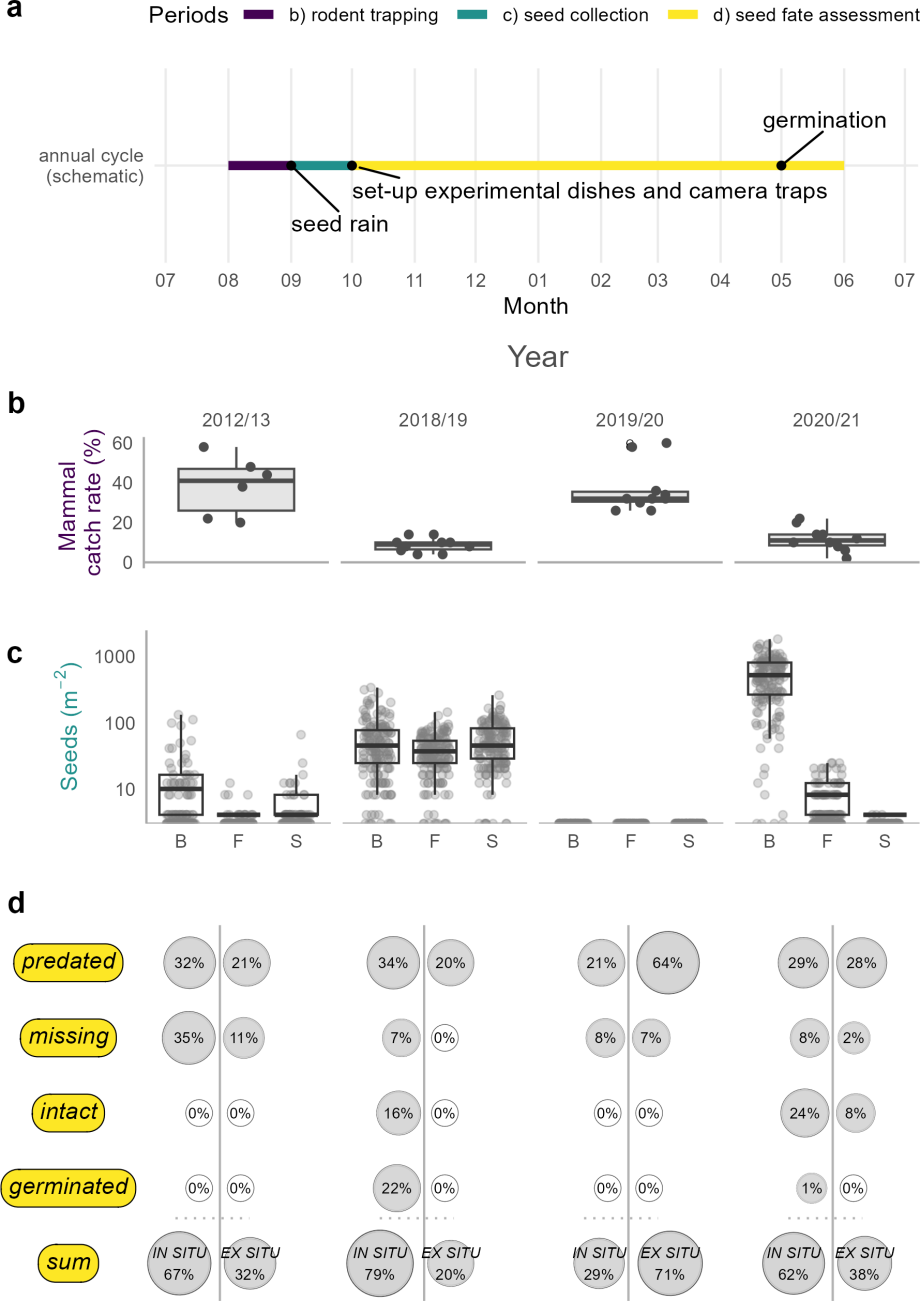
(*a*) Schematic overview of the annual fieldwork. (*b*) Percentage of occupied live traps by small mammals in August 2012 and 2018−2020. Boxplots represent the overall trap occupation rate by small mammals. The lower and upper hinges in (*b*) and (*c*) correspond to the first and third quartiles. Black dots represent the percentage of occupied traps for each of the two trapping grids during one trap night. A horizontal jitter reduces overlapping of shapes and enhances readability. (*c*) Number of seeds per square metre produced in 2012 and 2018−2020. Boxplots with additional dots representing seed traps (two 1 ha grids with 81 seed traps each). The three dominant tree species are separated and indicated with ‘B’ for European beech (*Fagus sylvatica*), ‘F’ for silver fir (*Abies alba*) and ‘S’ for Norway spruce (*Picea abies*). A horizontal jitter within each boxplot reduces overlapping of dots and enhances readability. The ordinate is log-transformed. (*d*) Final seed fate of European beech seeds produced in 2012 and 2018−2020. A bubble chart with rounded percentage represents the final fate of 639 individually tracked seeds from a cafeteria experiment classified in four categories: predated = seed predated, missing = seed fate unknown, intact = seed detected but neither germinated nor predated, germinated = seed germinated. A vertical bar separates seeds that were not dislocated until the final seed fate assessment in the following spring (i.e., left ‘*in situ*’) and dispersed seeds that have been found at least 10 cm from experimental dishes (*ex situ*).

Using long-term monitoring data, we determined the relationship between annual seed production and rodent abundance. Seed rain data were measured in two 1 ha study plots, each equipped with 81 seed traps arranged in a geostatistical design and we estimated the annual mean seed per square metre for the three dominant tree species (figure 1*c*, electronic supplementary material, S2) [27]. Small mammals were live-captured on two grids of 50 live traps for 3–5 consecutive trap nights in August [14,28]. The mammal catch rate is the ratio of occupied traps to the number of traps in a grid. We pooled two trapping grids with 50 traps each, and calculated the ratio for each trap night (figure 1*b*, see electronic supplementary material, S3 for the underlying data). The combination of both data sets provides a reliable estimate of the annual predator–prey ratio in the study area [28].

### (c) Cafeteria experiment and seed fate monitoring

To determine beech seed predation and hoarding behaviour of small rodents, we conducted cafeteria experiments in 2012/2013, 2018/2019, 2019/2020 and 2020/2021. In early October, we presented 18 dishes with 10 individually marked seeds, two of which were fitted with a VHF transmitter and eight with a 25 cm wire tag with a colourful flag (see electronic supplementary material, S4 for details about seed collection and arrangement and electronic supplementary material, S5 for the data). Due to minor differences in the set-up we used fewer seeds in 2012/2013 [14] (see electronic supplementary material, S6 6 for details about the setup of experimental dishes). Baseline germination rates were determined via germination tests with seeds collected in September 2018 and 2020 (electronic supplementary material, S7). To confirm the species identity of visiting rodents, we installed camera traps at each experimental dish in autumn 2019 and 2020 (see electronic supplementary material, S8 for details about camera trapping). Following presentation, we searched for tagged seeds at least once a week until weather prevented us from entering the study area (usually early to mid-November). After snowmelt in early spring (beginning of May), we collected remaining seeds, fragments and tags, assessed the final seed fate and conducted germination tests with seeds that did not germinate in the forest. In 2012/2013, the final seed fate in spring was recorded on the population level only (see electronic supplementary material, S9 for further interpretation). We noted whether a seed had been dispersed and categorized seed fate as intact, predated, missing or germinated. To visualize our results, we used R 4.4.1 [34] and the following R packages: tidyverse 2.0.0 [35,36], cowplot 1.1.3 [37], ggpattern 1.1.1 [38], ggtext 0.1.2 [39] and magick 2.8.4 [40].

## 3. Results

### (a) Seed rain and small mammal abundance

Seed production in beech, spruce and fir, as well as abundance of small rodents showed strong interannual variability and our study years covered the full range of seed-to-predator ratios (figure 1*b*,*c*). In 2012, seed production was low for all dominant tree species in our study area [27]. After moderate seed production in 2018 (65 seeds m^−^²), we observed a complete crop failure in 2019. Few conifer seeds were available in 2020, but European beech showed a peak in seed production (530 seeds m^−^²). Live trapping of small mammals revealed a pronounced abundance of *Myodes glareolus* in 2012 and of *Apodemus* spp. in 2019, accounting for 28.7% and 25.0% of occupied traps, respectively (figure 1*b*, electronic supplementary material, S3). The total trap occupation rate of small mammals during consecutive trap nights was highly variable between years: 38.3% in 2012, 8.8% in 2018, 36.6% in 2019 and 11.8% in 2020 (figure 1*b*, electronic supplementary material, S3).

### (b) Species composition of visitors at experimental dishes

Rodent species composition recorded at the experimental dishes varied between 2019 and 2020, but taxonomic groups other than small mammals were extremely rare in both years. In 2019, camera traps recorded 723 independent detections of small mammals (including 524 encounters of *Apodemus* spp. and 160 of *Myodes glareolus*) and in 2020, 79 small mammal detections (including 33 encounters of *Glis glis* and 13 of *Myodes glareolus*) during 15 and 23 days of operation, respectively [41]. Encounter rates on camera traps were in line with the species composition of live trapping and highlighted the enormous variation in visitation rates at experimental dishes between subsequent years. The only camera trap observation of a larger mammal was triggered by *Meles meles* in 2020. In addition to the frequent encounters with small mammals at the experimental dishes, we also detected two birds in 2019 and another in 2020.

### (c) Final seed fate

Rodent dispersal did not result in germinated seeds in our study (figure 1*d*). We tracked a total of 639 seeds, of which 536 (84%) could be located and assigned to a seed fate category beyond the seed stage. Rodents transported 20–71% of the seeds away from experimental dishes and not a single seed located *ex situ* germinated. The proportion of unrecovered seeds was variable between years and ranged from 7% to 15% in 2018−2020. The observed annual predation rate ranged from 53% to 85%. We only found intact dispersed seeds during the 2020/2021 trial, all other dispersed seeds were missing or depredated. Furthermore, tagging did not have aversive effects on the seeds, as shown by the 2018/2019 germination observations. Germination was successful only under natural conditions, as none of the visually intact seeds collected during the final assessment in spring germinated in the laboratory. Independent germination tests of additional seeds from our study site collected in 2018 and 2020 revealed baseline germination rates between 13 and 40% (see electronic supplementary material, S7).

## 4. Discussion

Our results, based on detailed tracking of seed fate with a high recovery rate (84% of provided seeds), support the hypothesis that seed handling by *Apodemus* and *Myodes* rodents does not result in effective seed dispersal for European beech at our study site. Across multiple years, which cover the range of potential seed rain and rodent abundance, not a single successful germinant was established from more than 500 seeds for which we tracked post-dispersal seed fate. In contradiction to the predator dispersal hypothesis, only seeds that were not transported by rodents germinated. Substantial germination (i.e. the emergence of a radicle) was exclusively found in 2018/2019 (22% *in situ*), a year with intermediate seed production, not a mast year. Seed predator populations in summer 2018 were low as seed production was very low in 2017 [28]. The high germination rate in 2018/2019 also led to a high input of seedlings in the study area [27]. Here, the prediction of the predator satiation hypothesis (higher germination rates in years of high seed production) is met. This is, however, not the case for the mast event in 2020/2021, a year with the highest beech seed production in 17 years [27] where only 1% of the exposed seeds germinated. We found very high predation rates during the mast event, which were only higher in the preceding year, when seeds were almost absent in our study area, which supports a nonlinear relationship between seed rodent ratio and seed fate [42].

Quantifying seed fate under field conditions is a technical challenge [14,43] and recent work has shown that subtle differences in field and statistical methods used to determine seed fate can result in vastly different estimates of rodent dispersal outcomes [15,44]. Most comparable studies focused on seed predation and removal but there is a lack of empirical data about seed fate tracked up to germination [7,26,43]. We used different tags to facilitate detection while maintaining their attractiveness to rodents and their germination capability and invented a wire loop bonding technique to ensure a stable attachment of visual markings without damaging the seed ([14], electronic supplementary material, S10). The tagging did not affect germination ability, as the maximum rate of germination from our experiment (22%) is well within the range observed under controlled laboratory conditions.

At our study site, seed rain of European beech peaks in September/October and germination could be observed in May or June. This period of approximately 8 months of tracking individual seeds is a demanding task, and we faced methodological challenges that provided context for our results, but did not affect the key inference: first, our study highlights the need to track seeds until they germinate or reach their biological expiration date, if we want to draw conclusions about the seed fate of the life stage of seeds. The extraordinarily high ratio of seed consumers and seeds in 2019, resulted in the highest rate of dispersal (71%), but ultimately ended with the highest rate of confirmed predation (85%), which is in line with findings from our previous work [11]. However, due to a complete lack of natural seed production in that particular year, we relied on commercial, sterilized seeds to conduct the experiments. Therefore, we could not rule out potential effects of differences in palatability on the results in the period 2019/2020. Second, wind, heavy rain and gliding snowpacks may have moved seeds downhill in our study area, potentially influencing our observations of short-distance dispersal. This, however, does not change the likelihood for germination, thus not influencing our key inference. All displaced seeds that survived winter were found on top of leaf litter within a radius of 0.5 m and were likely moved by snow, wind and rain. Camera trapping in 2019 and 2020 revealed that overall, three birds were recorded at our experimental sites, thus confirming that small mammals are the main interaction partners with seeds of European beech [33]. This finding is important, because the interaction frequency but not the quality of seed dispersal determines the net benefit for the plant partner [45,46]. That being said, with the animal partner in mind, it is worth noting that we had a single unusual observation of a red-toothed shrew *Sorex* sp. consuming a beechnut.

Our results illustrate that seed fate studies should span multiple cohorts, as the ecological context differs from year to year. Pronounced annual dynamics between seed production and subsequent abundances of granivores are a characteristic of masting species and are assumed to shift the position along the mutualism–antagonism continuum [2–5]. In years of high competition for seeds (i.e. low seed-to-rodent ratio in 2012/2013 and 2019/2020), we did not find a single intact or germinated seed, suggesting that high conspecific competition turns the rodent–beech interaction negative [4,12,26,47]. Even for mast years, predation rates of beechnuts by rodents vary considerably between studies ranging from 1% to 10% [48,49] up to rates as high as 61% to 74% [50] and our results confirm a pronounced reduction of seeds by rodent predation with more than 50% confirmed predation in each year of the study. From the plant's perspective, however, it is more important how many seeds successfully germinate. This result is further supported by our previous finding that local seed rain, without additional secondary dispersal but in combination with light conditions, is the main driver of recruitment [27]. Spatiotemporal dynamics of both interaction partners do not allow for an absolute negation of mutualistic interactions. For instance, some rodent species accumulate in medium-sized canopy openings following mast events [28], but the overall impact of disturbance dynamics on seed–disperser interactions in our study system is still unknown. Additionally, we acknowledge that our sample size, although larger than for many studies, is still limited compared with the large number of available seeds. After mast events, several hundred beechnuts cover each square metre of the forest floor [27] and the ratio between seed production and germination or even plant establishment is probably exceedingly small [51]. We cannot rule out that positive interactions occur, but they are likely rare and thus remain unobserved.

By tracking the fate of individual seeds until the end of the seed stage, our study provides relevant information and contributes to our understanding of the seed dispersal loop [52]. We found intact or germinating seeds only after intermediate and high seed production events in 2018 and 2020, but recruitment was stronger after the intermediate seed production event in 2018 than after the largest seed production event. This finding partially contradicts the predator satiation hypothesis, supporting our previous finding that economy-of-scale seed fate effects are complex and context-dependent [27,53]. Several studies showed positive effects of intermediate seed–rodent ratios and nonlinear species interactions were supposed to maintain ecosystem functions by balancing positive and negative effects for both interaction partners [42,54,55]. In this study, we did not observe any germinants from dispersed seeds and our results do not support the predator dispersal hypothesis. Seeds that are buried in shallow caches have better chances to escape predation [56] and are able to endure late frost or drought events [26,57,58] but our results provide empirical evidence that this is not always a precondition. In the trial 2018/2019, we noted a germination rate of 22%, although none of the seeds were buried but situated on top of the leaf litter. In contrast, 32% of non-damaged seeds did not germinate in the trial 2020/2021, while germination rate under laboratory conditions was 25.75% (electronic supplementary material, S7). We occasionally detected seeds larder-hoarded in deep burrows and under tree trunks and we assume that at least some of the missing seeds have been placed deep in the soil with no chance of germination.

In summary, our findings show mostly antagonistic interactions between rodents and seeds of European beech, which is in line with findings on other plant species [59,60]. The position of the interaction along the continuum between antagonism and mutualism is context-dependent and affected by the dynamics of both partners. The dynamics in seed production of European beech is affected by climate change, leading to an increased seed production but less annual variation [61]. As masting behaviour of European beech is a main driver for population dynamics of granivorous rodents in temperate forests [28] the position along the mutualism–antagonism continuum might shift in the long term, highlighting the value of ongoing research on plant–animal interactions. We can only speculate that dampened masting patterns and less frequent mast failures translate into more stable rodent populations among years and increased seed predation rates in years with sporadic seed production. In contrast, climate change, emerging diseases and other factors might result in a decoupling of rodent population cycles and mast seeding, with potential effects for beech seed survival and a less pronounced interaction between rodents and European beech.

## Data Availability

Camera trap data is available from the Dryad Digital Repository [41]. Other data are included within the electronic supplementary material. A description of each data set is provided within the same document as the data. Seed rain data: see supplementary material, S2; live trapping data: see supplementary material, S3; seed fate data: see supplementary material, S5; germination tests data: see supplementary material, S7. Supplementary material is available online [62].
